# *Epidermal Growth Factor Receptor* Gene Mutation Detection in Histology and Cytology Specimens of Primary Lung Adenocarcinoma: Immunohistochemistry Versus the Molecular Method

**DOI:** 10.31557/APJCP.2021.22.6.1935

**Published:** 2021-06

**Authors:** Nurul Shuhada Abdul Hamid, Anani Aila Mat Zin

**Affiliations:** *Pathology Department, School of Medical Sciences, Universiti Sains Malaysia, Kota Bharu, Kelantan, Malaysia. *

**Keywords:** Epidermal growth factor receptor, E746-A750 deletion, immunohistochemistry, lung adenocarcinoma

## Abstract

**Background:**

*Epidermal growth factor receptor* (*EGFR*) gene in lung adenocarcinoma is associated with good clinical response to EGFR-tyrosine kinase therapy. The two most common *EGFR* gene mutations, representing 80 to 90%, are the E746-A750 deletion in exon 19 and the L858R point mutation in exon 21.

**Materials and Methods:**

We have conducted the study to evaluate immunohistochemistry’s performance in detecting the E746-A750 deletion in exon 19 of the *EGFR* gene in primary lung adenocarcinoma cases. This study examined 133 cases of primary lung adenocarcinoma for three years duration. The selected cases were tested for *EGFR* gene mutations by real-time PCR by a reference laboratory. Most cases (124) were diagnosed by tissue biopsy, though nine used cell block cytology. We performed an immunohistochemistry test on 75 cases that contained adequate diagnostic material in the paraffin block.

**Results:**

The test result was scored as 0 to 3+, based on the staining intensity and percentage of positive tumor cells. We evaluated the immunohistochemistry test’s sensitivity and specificity compared to the *EGFR* gene mutations by real-time PCR. There was a significant association between gender, smoking status, and the *EGFR* gene mutations (P < 0.001). The overall sensitivity and specificity of the immunohistochemistry test were 40% and 100%, respectively. The positive predictive value and negative predictive values were 100% and 76.9%, each.

**Conclusions:**

The immunohistochemistry has high specificity but low sensitivity in the detection of E746-A750 deletion in exon 19 of the* EGFR* gene. The mutation-specific antibody used in this study was unable to detect other uncommon variants of exon 19 deletions. With high specificity value, immunohistochemistry may provide an adjunct to molecular testing for detecting the most common *EGFR* gene mutations in cases of a low cellularity sample, financially-limited situations, or in critically ill cases where urgent targeted therapy is needed.

## Introduction

Lung cancer remains the most common cancer worldwide, with an estimated 2.09 million cases reported in the year 2018, and is also associated with the highest cancer-related deaths (Torre et al., 2012; Bray et al., 2018). Based on the national data, lung cancer is the second most common cancer in males and the fifth most common cancer in females. The peak age of diagnosis is 70 and above, and more frequently encountered among Malay populations. At diagnosis, most cases are inoperable, locally advanced, or metastatic disease (Azizah et al., 2016). According to the Malaysian study on cancer survival, 3,007 cases of stage IV lung cancer were diagnosed from 2007 to 2011, with a five-year survival of only 6.3 % (Azizah Noor Hashimah et al., 2018). Lung cancer diagnosis requires a combination of clinical evaluation, radiology, and pathological findings. Recent studies have shown that the incidence of lung adenocarcinoma is increasing, but squamous cell carcinoma is decreasing (Alberg et al., 2013; Liam et al., 2006). There are several driver gene mutations in lung carcinoma pathogenesis, including *EGFR, KRAS, BRAF, HER2, AKT1, NRAS, PIK3CA, MEK1, EML4-ALK*, and MET amplification. The most relevant mutations in clinical practice are *EGFR* gene mutations and ALK rearrangements. These two mutations are usually seen in many tumour especially lung adenocarcinoma, and there are specific targeted therapies available (Lindeman et al., 2018; Tsao et al., 2006). Epidermal growth factor receptor (EGFR) mutations are observed in a big proportion of patients with non-small cell lung cancer (NSCLC) from Asian populations (Lee et al., 2021). Overexpression of *EGFR* is also seen in gallbladder carcinoma which suggesting that altered expression of these genes maybe a possible mechanism in carcinogenesis (Abee et al., 2021). Based on the Izadian et al., (2017), *CDH1* and *EGFR *biomarkers increased in the black lesions (anthracosis) of lung tissue. According to the latest guidelines by the American College of Pathologists (2018), reflex molecular testing of *EGFR* gene mutations by next-generation sequencing method should be performed in all cases of primary lung cancer with adenocarcinoma component (Lindeman et al., 2018). 

Although molecular testing is the gold standard for detecting *EGFR* gene mutations, the facilities are not readily available in most pathology laboratories in Malaysia. Molecular testing is expensive, complicated, and requires specialized personnel to operate and interpret the test. Guidelines recommended first-line (1L) treatment of EGFR mutations advanced NSCLC with first- or second-generation (1G/2G) EGFR-tyrosine kinase inhibitors (TKIs) erlotinib, gefitinib or afatinib (Lee et al., 2021). Because of the importance of identifying the *EFGR* gene mutation, particularly in lung adenocarcinoma, we conducted this study to evaluate immunohistochemistry’s performance in detecting the specific EGFR mutated protein. Immunohistochemistry is a well-established test and routinely performed in all anatomical pathology laboratories. It is a rapid one-day process and cheaper compared to molecular testing. The test can be performed in a case where limited diagnostic material available such as cell block cytology, in which the tumor concentration may be below the sensitivity level detected by a molecular test (Chabot-Richards et al., 2015). Also, most pathologists are familiar with immunohistochemistry and can interpret the result without difficulty. The aim of this study was to evaluate immunohistochemistry’s performance in detecting the E746-A750 deletion in exon 19 of the *EGFR *gene in primary lung adenocarcinoma cases.

## Materials and Methods

*Case and sample selection*

This cross-sectional study was carried out at Hospital Raja Perempuan Zainab II, Kota Bharu, for 42 months from January 2014 to June 2017, including 133 cases. The selected cases were primary lung adenocarcinoma, diagnosed based on tissue biopsy (histology) or cytology samples. In this study, we further categorized the histologic diagnosis into NSCC favor adenocarcinoma, NSCC NOS, favor adenosquamous carcinoma, and NSCC NOS according to the recent 2015 WHO classification of a lung tumor in small biopsy and cytology samples. Clinical and pathologic data were obtained by reviewing medical and pathology reports. Tumor staging was based on the clinical stage because there was no resected specimen available upon diagnosis. All 133 cases were tested for the *EGFR* gene mutations by real-time polymerase chain reaction (real-time PCR) by a reference laboratory. We found 75 cases from tissue biopsy and cell block cytology with sufficient material for the immunohistochemistry test. The sample size was calculated by using the estimation of sensitivity and specificity using sample size calculator version 1.7 from Unit Biostatistics and Research Methodology, Universiti Sains Malaysia. 

*Immunohistochemistry procedures*

According to the standard laboratory protocol and manufacturer guidelines, we performed the immunohistochemistry test using a semi-automated method. The immunohistochemistry used was a mutation-specific rabbit monoclonal antibody, clone SP111, to detect detection of E746-A750 deletion in exon 19 of the *EGFR* gene. Briefly, 3 to 4-micron thick sections of tissue were transferred to poly-l-lysine precoated slides. After placed on a hot plate at 60ºC for 1 hour, the slides were rehydrated with two changes of xylene for 5 minutes each, followed by immersion in two changes of absolute alcohol (100%) for 2 minutes each. The slides were immersed into four different descending concentrations of alcohol for 2 minutes each, subsequently in distilled water for 2 minutes. Heat-induced epitope retrieval was applied for antigen retrieval by using the pressure cooker method. The slides were incubated in antigen retrieval buffer (Tris/EDTA pH 9.0, sodium citrate pH 6.0) within a fully pressurized pressure cooker for 3 minutes, followed by the process of cooling down in cold running water for 10 minutes. The application of peroxidase blocking agent was using Squenza Immunostainer, incubated for 5 minutes, and followed by a brief washed in distilled water. The primary antibody dilution was 1: 100, incubated overnight at 4ºC, followed by washing in Tris-buffered tween solution (Kitamura A, Hosoda W, Sasaki E, Mitsudomi T, Yatabe Y et al., 2010). The slides were then incubated in 3,3’-diaminobenzidine for 5 minutes and washed with distilled water, followed by counterstaining with Harris Hematoxylin for 5 seconds, dehydration, and coverslipped using Cytoseal XYL mounting medium. 

*Immunohistochemistry scoring*

The immunohistochemistry scoring was modified according to the previous literature (Seo et al., 2014; Hitij et al., 2017; Brevet et al., 2010). The evaluation was performed under high power magnification (400x). The scores were based on the staining intensity of cytoplasmic and/or membranous distribution and percentage of positive tumor cells, as listed below: 

• Score 0: No staining or weak staining, in <10 % of tumor cells 

• Score 1+: Weak staining, in ≥ 10% of tumor cells 

• Score 2+: Moderate staining, in ≥ 10% of tumor cells 

• Score 3+: Strong staining, in ≥ 10 % of tumor cells 

Scores of 1 and above were considered a positive result (Hitij NT, Kern I, Sadikov A, et al., 2017); Brevet M, Arcila M, Ladanyi M. et al., 2010). The test evaluation was performed by a senior pathologist, who was blinded to the clinical history and the *EGFR *gene mutations result by real-time PCR. 

*EGFR gene mutations by real-time PCR*

Data for *EGFR* gene mutations result of exon 18 to 21 by real-time PCR performed by a reference laboratory (Subang Jaya Medical Center) were retrieved from the Department of Pathology, Hospital Raja Perempuan Zainab II, Kota Bharu. 

*Statistical analysis*

Descriptive statistics were used to describe the clinicopathological data. The association between clinicopathological data and the *EGFR* gene mutations was calculated using the Pearson Chi-Square test or Fisher’s exact test when appropriate. A p-value of <0.05 indicated statistical significance. The sensitivity and specificity of the immunohistochemistry test compared to the real-time PCR were calculated. All statistical analyses were conducted using Statistical Package for Social Sciences (SPSS) program version 24.

## Results

*Clinicopathological data*

Table 1 summarizes the clinicopathological data of the selected cases. The 133 cases consisted of 73 males (54.9%) and 60 females (45.1%), with a mean age of 59.5. Malay ethnicity is predominant, 123 cases (92.5%), followed by Chinese, 9 cases (6.8%), and 1 Siamese (0.8%). There were 66 patients (49.6%) who were never smokers, while 12 (9.0%) were smokers. Ex-smokers accounted for 47 cases (35.3%). Smoking history was not available in 8 cases (6.0%). The clinical staging was assigned according to the 8th edition of the American Joint Committee on Cancer TNM classification. By the TNM stage, the majority, 128 cases (96.2%) were diagnosed at stage IV with only 5 cases (3.8%) at stage III, with none diagnosed as Stage I or II. We diagnosed 124 cases (93.2%) based on the tissue biopsy, with the cytological diagnosis of 9 cases (6.8%). NSCC, favor adenocarcinoma was predominant, 124 cases (93.2%), followed by NSCC, NOS of 4 cases (3.0%), and 5 cases (3.8%) of NSCC, NOS possible adenosquamous carcinoma. *EGFR* gene mutations were present in 59 cases (44.4%) and absent in 74 cases (55.6%). Exon 19 deletion was detected in the majority of cases involving 44 cases (33.1%), followed by exon 21 mutation in 13 cases (9.8%), with only one each (0.8%) for exon 18 and exon 20 mutations. Two cases (1.5%) with exon 19 deletion on the initial biopsy developed disease progression following tyrosine kinase therapy, and a repeat molecular testing showed acquired T790M mutation, which was not detected before treatment given.

*Association between clinicopathological data and the EGFR gene mutations by real-time PCR*

Table 2 presents the association between the clinicopathological data and the *EGFR* gene mutations. There was a statistically significant association between gender and *EGFR* gene mutations. Mutations occurred more frequently in females than males (73.3% versus 20.5%, P < 0.001). There was no significant association between mean age and the detection of the *EGFR* gene mutations (P = 0.599). There were higher mutation rates among Malay (44.7%) than Chinese (44.4%), and there was no mutation in one Siamese case. However, the difference was not statistically significant (P > 0.950). There was no significant association between clinical staging and the *EGFR* gene mutations. We further analyzed cases with known smoking status. The *EGFR* gene mutations were more common in never-smoker’s cases (69.7%) than in ever-smokers (15.3%). 

The association was statistically significant, with P < 0.001 (Table 3). Most female (56 cases, 96.6%) were never smokers, while the majority of the males were either ex-smoker or current smoker (57 cases, 85.1%). There was no significant association between each type of exon mutation with smoking history, with P > 0.05 (Table 4). 

*Immunohistochemistry results*

An immunohistochemistry test was performed in 75 cases (56.4%). The remaining 58 cases (43.6%) had exhausted tissue in the paraffin block, thus unable to proceed with the test. 

**Figure 1:**
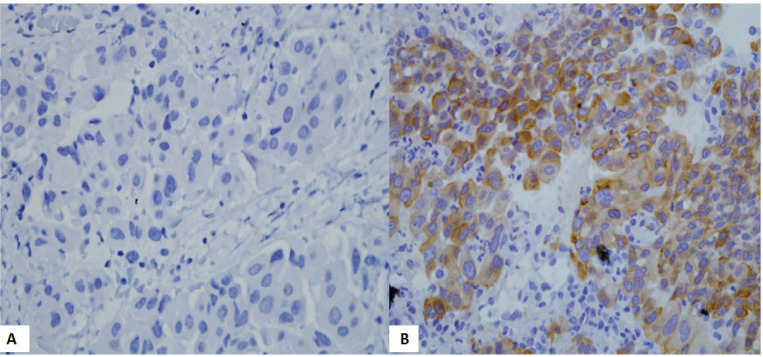
Immunohistochemistry Test for the E746-A750 Deletion in Exon 19 of the EGFR Gene. (A) negative control (x400 magnification), (B) positive control (x400 magnification)

Total of 65 cases (48.9%) showed no immunohistochemistry expression (score 0). Ten cases were positive for immunohistochemistry, with a score of 1+ (4 cases), 2+ (4 cases), and 3+ (2 cases). Out of 65 cases with a score of 0 on immunohistochemistry, 49 cases had negative *EGF*R gene mutations by real-time PCR. One case with exon 21 mutation detected by real-time PCR also showed a score of 0 on immunohistochemistry. However, in the remaining 15 cases, exon 19 deletion was detected by real-time PCR. Ten cases with positive immunohistochemistry tests were confirmed to have exon 19 deletions of the *EGFR* gene by the real-time PCR (Table 5). Figure 1 showed immunohistochemistry results according to each score. 

*Sensitivity and specificity of the immunohistochemistry expression*

The sensitivity, specificity, PPV, and NPV were calculated and presented in Table 6. Score 1 and above were considered positive. The immunohistochemistry showed excellent specificity results (100%), with no false-positive results detected compared to real-time PCR. However, the test had low sensitivity (40%). There were fifteen cases of false-negative immunohistochemistry, which contributed to the low sensitivity result. The PPV was excellent (100%), indicates that in a case where the immunohistochemistry test was positive, there is a high probability that the patients harbor *EGFR* gene mutations. The NPV was fair, 76.9%. In cases with negative immunohistochemistry results, a confirmatory *EGFR* gene mutation by a molecular method is strongly indicated.

**Table 1:** Clinicopathological Data and the EGFR Gene Mutations by Real-Time PCR (number = 133)

Variables	number (%)
Sex	
Male	73 (54.9)
Female	60 (45.1)
Age	59.52 (11.01)
Ethnicity	
Malay	123(92.5)
Chinese	9 (6.8)
Others	1 (0.8)
Smoking Status	
Smoker	12 (9.0)
Never-smoker	66 (49.6)
Ex-smoker	47 (35.3)
Not available	8 (6.0)
Clinical Staging	
Stage I	0 (0.0)
Stage II	0 (0.0)
Stage III	5 (3.8)
Stage IV	128 (96.2)
Type of Specimen	
Biopsy/Histology	124(93.2)
Cytology	9 (6.8)
Histological Diagnosis	
NSCC, favor ADC	124 (93.2)
NSCC, NOS	4 (3.0)
NSCC, NOS possible	5 (3.8)
adenosquamous	
EGFR mutation analysis	
Wild-type (negative)	74 (55.6)
Exon 18	1 (0.8)
Exon 19	44 (33.1)
Exon 20	1 (0.8)
Exon 21	13 (9.8)
Acquired T790M mutation after	
treatment	2 (1.5)

EGFR, Epidermal Growth Factor Receptor; PCR, Polymerase Chain Reaction.

**Table 2:** Association between Clinicopathological Data and the EGFR Gene Mutations by Real-Time PCR (n = 133)

	EFGR gene mutation status			
Variables	Negative	Positive	number	p-value
	number (%)	number (%)		
Gender				<0.001^a^
Male	58 (79.5)	15 (20.5)	73	
Female	16 (26.7)	44 (73.3)	60	
Age				0.599^a^
≤ 60 years old	38 (53.5)	33 (46.5)	71	
>61 years old	36 (58.1)	26 (41.9)	62	
Ethnicity				>0.950^b^
Malay	68 (55.3)	55 (44.7)	123	
Chinese	5 (55.6)	4 (44.4)	9	
Others	1 (100.0)	0 (0.0)	1	
Clinical Staging				0.382^b^
Stage III	4 (80.0)	1 (20.0)	5	
Stage IV	70 (54.7)	58 (45.3)	128	

^a^Chi-Square test, bFisher’s Exact test; EGFR, Epidermal Growth Factor Receptor; PCR, Polymerase Chain Reaction

**Table 3:** Association between Smoking Status and the EGFR Gene Mutations (number = 125)

Variables	EFGR gene mutation status		number	P-value (Chi-square)
	Negative number (%)	Positive number (%)		
Smoking status				<0.001
Ever-smokers	50 (84.7)	9 (15.3)	59	
Never-smokers	20 (30.3)	46 (69.7)	66	

EGFR, Epidermal Growth Factor Receptor

**Table 4:** Association between Specific Exon Mutations of the EGFR Gene and Smoking Status (number = 55)

Variables	Smoking status		number	aP-value
	Ever-smoker	Never-smoker		
	number (%)	number (%)		
Exon				
18	0 (0.0)	1 (100.0)	1	1
19	8 (19.5)	33 (80.5)	41	0.421
20	0 (0.0)	1 (100.0)	1	1
21	1 (8.3)	11(91.7)	12	0.666

^a^, Fisher’s Exact test; EGFR, Epidermal Growth Factor Receptor

**Table 5:** Immunohistochemistry of EGFR Expression in Comparison with the Real-Time PCR Method (Number = 75)

	Immunohistochemistry expression (score / number)	Real-time PCR analysis (number)
Exon 18	-	-
		
Exon 19	Score 0 (15)	25
	Score 1+ (4)	
	Score 2+ (4)	
	Score 3+ (2)	
Exon 20	-	-
Exon 21	Score 0 (1)	1
Wild type	Score 0 (49)	49

EGFR, Epidermal Growth Factor Receptor; PCR, Polymerase Chain Reaction

**Figure 2:**
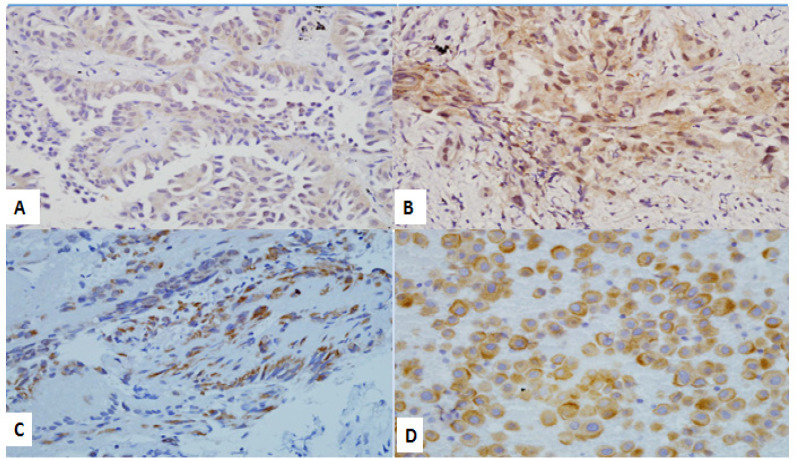
Immunohistochemistry Result for the E746-A750 Deletion in Exon 19 of the EGFR Gene. (A) tissue biopsy, score 1+, weak staining in ≥ 10% of tumor cells (x400 magnification), (B) tissue biopsy, score 2+, moderate staining, in ≥ 10% of tumor cells (x400 magnification).(C) tissue biopsy,score 3+, strong staining, in ≥ 10 % of tumour cells (x400 magnification), (D) pleural fluid cytology (cell block), score 3+ (x400 magnification).

**Table 6:** Sensitivity, Specificity, PPV, and NPV of Immunohistochemistry Test in the Detection of the EGFR Gene Mutations

	Percentage	95% CI
Sensitivity	40	34.3- 45.6%
Specificity	100	99.0-100.0%
Positive predictive value	100	99.0- 100.0%
Negative predictive value	76.9	72.0- 81.8%

Abbreviations: PPV, positive predictive value; PPV, number of positive; cases / number of cases with positive screening results × 100,000; NPV, negative predictive value; NPV, number of negative; cases / number of cases with positive screening results × 100,000

## Discussion

Lung cancer is the third most common cancer in Malaysia, based on the National Cancer Registry (NCR) published in 2016. According to the national statistics, for males, lung cancer is the second most common cancer accounted for 7,415 cases, and the fifth most common cancer in the female with 3,193 cases reported in 5 years (Azizah et al., 2016). In our study, there was no significant gender difference between the number of cases reported (73 males and 60 females). The majority of the Kelantan population are Malay, thus explained the highest frequency of lung adenocarcinoma among Malay ethnicity in this study. Smoking-related lung carcinoma was well-described in the literature (Liam et al., 2006). Interestingly, based on our data, almost half of lung adenocarcinoma cases with *EGFR* gene mutations were female with no tobacco exposure. Sun et al., (2007) defined a never-smoker as an individual who has had less than 100 cigarette exposure in a lifetime. Lung cancer in never-smokers has distinct epidemiological data, clinical manifestations, and histological subtype (Yang et al., 2011). Our findings are similar to those previously reported that never-smoker lung cancer occurs more frequently in women and East Asians, usually involving distal airways with the most common histological subtype of adenocarcinoma (Sun al., 2007; Yang et al., 2011; Liu et al., 2000; Liam et al., 2013). The pathogenesis of lung cancer in never-smoker are not well-understood. Several studies described the relationships between second-hand smoke, radon exposure, indoor air pollutants from cooking oil, and the smoke from burning charcoal, with the occurrence of lung cancer in never-smokers (Yang et al., 2011; Sun et al., 2007; Wakelee et al.,2007). The role of estrogens and other female hormones in the development of lung cancer in the female, never-smoker is uncertain and controversial. Wu et al., (2005) discovered that the expression of the estrogen-receptor beta was more common in a non-small cell lung carcinoma in never-smoker compared to the smoker. Based on in vitro study by Márquez-Garbán et al., (2009), estrogen stimulates the growth of human non-small cell lung carcinoma. However, a randomized controlled trial by Chlebowski et al., (2010) showed there was no increase in the risk of lung cancer and mortality rate in women who received estrogen compared to placebo. We also found out that the frequency of* EGFR* gene mutations in lung adenocarcinoma was higher in the never-smoker (69.7%) compared with the patient with previous tobacco exposure (15.3%), which was concordant with other previous studies. Driver genes that are involved in lung carcinogenesis are *EGFR, KRAS, BRAF, HER2, AKT1, NRAS, PIK3CA, MEK1, EML4-ALK*, and* MET *amplification (Lindeman et al., 2018). One of the most significant molecular differences in never-smoker and smoker lung adenocarcinoma is *EGFR* gene mutations. *EGFR* gene mutations are seen in up to 40% of never smoker, compared to 1% in the smoker, while KRAS mutation is almost exclusively limited to smoker’s lung cancer (Yang et al., 2011; Sun et al., 2007; Sonobe et al., 2005; Travis et al., 2015). EGFR is a transmembrane receptor tyrosine kinase that regulates normal cell survival and cell development. In the mutated form of the tyrosine kinase domain, the receptor activity is dysregulated and allows cancer cells to proliferate and survive (Herbst et al., 2008). Several drugs have been developed to target the receptor activity specifically. *EFGR* gene mutations lung adenocarcinoma in never-smoker has shown higher response rate and better survival if treated with EGFR tyrosine kinase targeted therapy, compared to smoker or ex-smoker (Pao et al., 2004; Shepherd et al., 2005; Tsao et al., 2005). In our study, the excellent performance of immunohistochemistry was identified in terms of specificity and PPV (100%, respectively); however, it has low sensitivity and NPV (40% and 76.9% each). Through various literature searches, our result showed a similar trend with the previous studies. The specificity value of immunohistochemistry was reported as 96.1 to 100%, with a wide range of sensitivity levels, from 40% to 94.1% (10-13, 28). The antibody used in this study was specific in the detection of E746-A750 deletion in exon 19 of the *EGFR* gene. No false-positive result was observed in the patient with exon 21 mutation or wild-type. Because of the high specificity rate, the immunohistochemistry test is reliable and could be performed in selective conditions, as discussed below. In Asian countries, the documented rate of *EGFR* gene mutations is higher compared to Western populations (Liam et al., 2013). Thus, all lung adenocarcinoma cases in Malaysia should be tested for *EGFR* gene mutations. There are only a few recognized molecular laboratories in this country that routinely performed *EGFR* gene mutations testing. The molecular test is not readily available and accessible at all times. Immunohistochemistry can be used in cases where molecular testing cannot be performed due to financial limitations. Furthermore, in cases with suboptimal tissue available for DNA amplification, either due to low tumor contents or decalcified tissue from bone metastasis, immunohistochemistry can be performed as a screening tool for the detection of the *EGFR* gene mutations. Immunohistochemistry is also recommended in the case of a critically-ill patient where urgent identification of the *EGFR* gene mutations and early initiation of targeted therapy are strongly needed. Immunohistochemistry can provide faster results than the PCR-based method may require at least 10 to 21 days to completion of the test. Immunohistochemistry performance, according to our data, showed low sensitivity and NPV value. We used a commercially available mutation-specific antibody to detect exon 19 deletion, involving only E746-A750 deletion. The selection of the antibody was based on the local data by Liam et al., (2013), which reported that the most common *EGFR* gene mutations in Malaysia were exon 19 (23.5%) followed by exon 21 (14.9%). Another study by Roengvoraphoj et al., (2013) showed up to 80 to 90% of the *EFGR* gene mutations in lung adenocarcinoma are short in-frame deletions in exon 19 and a specific point mutation in exon 21 at codon 858. Cheng et al., (2012) described there are over 20 variants of exon 19 deletions, with the most common, including E746-A750 deletion, L747-T751insS deletion, and L747-P753insS deletion. Thus, the lower sensitivity result of the immunohistochemistry test in our study was not surprising. The antibody was likely unable to detect other variants of exon 19 deletions. To overcome this, the application of multiple antibody panels, which can detect other variants of exon 19 deletions, is another method to increase the sensitivity level. However, the use of multiple antibodies will consume more tissue and not cost-effective. Based on the low sensitivity and NPV, immunohistochemistry is inferior compared to the standard molecular method for *EGFR* gene mutation testing. Patients with a negative immunohistochemistry test still have a high probability of harboring *EGFR* gene mutations based on our findings; thus, a confirmatory test by a molecular method is mandatory. The major limitation of this study was during the pre-analytical phase. There were 58 cases with scanty diagnostic material, inadequate for further immunohistochemistry testing. The majority of cases were diagnosed as Stage III and IV, with no resected specimen received, and diagnostic material was mainly from a small tissue biopsy and cytology. The same specimen was subjected for routine H&E staining, immunohistochemistry test for differentiation of either squamous or adenocarcinoma, and molecular testing to a reference molecular laboratory. The latter test requires at least ten unstained slides. Thus, only a minimal amount of tissue left in the paraffin blocks for the research purpose. Based on the recommendation by Nor Salmah B, Mardiana AA, Ruzi H, Rose A, Noor Kaslina MK et al.,(2018), for tissue optimization in lung carcinoma cases, they proposed multiple levels of the tissue as follows; Level-1 for H&E, followed by two unstained slides on poly-l-lysine, Level-2 H&E, another two unstained slides on poly-l-lysine, six unstained slides for *EGFR* gene mutations testing, and Level-3 for H&E. We believed that if we applied a standardized protocol for tissue utilization during the initial diagnostic process, more tissue would be available for future research work. Also, the optimization process of the primary antibody was challenging. The main issue was to identify the ideal dilution and incubation time. Based on the manufacturer’s guidelines, the primary antibody dilution was 1:100 with 1-hour incubation time. We were unable to detect any positive cells during several trials according to the suggested protocol. We did multiple literature searches, but most of the previous studies used an automated method, while we were using a semi-automated technique. We modified a few techniques applied by different authors (Kitamura et al., 2010; Brevet et al., 2010), and finally managed to get a satisfactory result. Due to multiple trials for the optimization process, we had limited primary antibody to test on the tissue samples.

In conclusion, the immunohistochemistry test has shown excellent specificity, however, with low sensitivity in the detection of the most common *EGFR* gene mutation in a series of primary lung adenocarcinoma cases. Although a low sensitivity rate was observed, the test can be used in certain conditions such as critically-ill cases that require urgent tyrosine kinase therapy, low cellularity, and poor-quality samples and in financially-limited conditions. Immunohistochemistry is a rapid test, easily interpreted, and cost-effective, but its role is limited since it is unable to detect other variants of the *EGFR* gene mutations. Overall, immunohistochemistry can be used as an adjunct to the molecular method in identifying the *EGFR* gene mutations in lung adenocarcinoma. 

## Author Contribution Statement

Nurul Shuhada:runned the labwork, collected data and analyzed data. Anani Aila: designed study,edited final version and reviewed the paper. All authors read an approved the final version. 
